# Los recursos humanos en salud según el nuevo modelo de atención en Ecuador

**DOI:** 10.26633/RPSP.2017.52

**Published:** 2017-05-15

**Authors:** Verónica Espinosa, Daniel de la Torre, Cecilia Acuña, Cristina Cadena

**Affiliations:** 1 Ministerio de Salud Pública del Ecuador Viceministerio de Gobernanza y Vigilancia de la Salud Quito Ecuador Ministerio de Salud Pública del Ecuador, Viceministerio de Gobernanza y Vigilancia de la Salud.; 2 Organización Panamericana de la Salud Organización Panamericana de la Salud Quito Ecuador Organización Panamericana de la Salud, Quito, Ecuador.

**Keywords:** Reforma de la atención de salud, atención de la salud, atención integral de salud, recursos humanos, Ecuador, Health care reform, health care (public health), comprehensive health care, human resources, Ecuador

## Abstract

**Objetivo.:**

Describir las estrategias implementadas por el Ministerio de Salud Pública del Ecuador (MSP) para fortalecer la rectoría de los recursos humanos en salud y responder al nuevo modelo de atención, como parte del proceso de reforma durante el periodo 2012–2015.

**Métodos.:**

Se realizó una revisión documental sobre el desarrollo del recurso humano en salud antes y después de la reforma recabando información en fuentes primarias y secundarias.

**Resultados.:**

En el periodo de estudio, Ecuador generó un nuevo marco institucional y normativo para desarrollar recursos humanos en salud a fin de responder a los requisitos de un modelo de atención basado en la Atención Primaria en Salud (APS). El MSP consolidó su papel rector estableciendo alianzas estratégicas, aplicando métodos de planificación de los recursos humanos y realizando una inversión sin precedentes en la formación del personal sanitario, contrataciones e incrementos salariales. Estos elementos constituyen los ejes iniciales para la construcción de una política de recursos humanos en salud y una carrera sanitaria coherentes con los objetivos de la reforma.

**Conclusiones.:**

En el marco de la reforma realizada entre 2012 y 2015, el desarrollo del recurso humano en salud muestra logros importantes gracias al trabajo intersectorial realizado por el MSP. Entre dichos logros destacan el fortalecimiento de la rectoría, el desarrollo e implementación de normativa e instrumentos regulatorios, la creación de nuevos perfiles de profesionales, y el contrato de profesionales con el objetivo de implementar el Modelo de atención integral de salud (MAIS), lo cual contribuyó a resolver problemas arrastrados durante los años previos a la reforma.

## Abstract (en)

**Objective.:**

Describe strategies implemented by Ecuador’s Ministry of Public Health (MPH) to strengthen human resources for health leadership and respond to the new model of care, as a part of the reform process in the period 2012–2015.

**Methods.:**

A documentary review was carried out of primary and secondary sources on development of human resources for health before and after the reform.

**Results.:**

In the study period, Ecuador developed a new institutional and regulatory framework for developing human resources for health to respond to the requirements of a model of care based on primary health care. The MPH consolidated its steering role by forging strategic partnerships, implementing human resources planning methods, and making an unprecedented investment in health worker training, hiring, and wage increases. These elements constitute the initial core for development of human resources for health policy and a health-services study program consistent with the reform’s objectives.

**Conclusions.:**

Within the framework of the reform carried out from 2012 to 2015, intersectoral work by the MPH has led to considerable achievements in development of human resources for health. Notable achievements include strengthening of the steering role, development and implementation of standards and regulatory instruments, creation of new professional profiles, and hiring of professionals to implement the comprehensive health care model, which helped to solve problems carried over from the years prior to the reform.

La promulgación de una nueva Constitución del Ecuador en 2008 generó una serie de cambios y reformas a nivel institucional, administrativo y jurídico (1). En este contexto, diversos organismos estatales pusieron en marcha los procesos de reestructura requeridos por el mandato Constitucional, lo cual determinó un nuevo escenario en el cual el Ministerio de Salud Pública (MSP) debía hace frente a nuevos y antiguos retos.

En este marco, el MSP recibió el mandato explícito de asumir el papel rector en el ámbito de la salud, incluyendo el de fortalecer los servicios públicos de salud e incorporar recursos humanos cualificados y en número suficiente para brindar una atención de calidad y con calidez.

## MATERIALES Y MÉTODOS

En este artículo se utiliza un método descriptivo basado en la revisión documental de fuentes primarias del Ministerio de Salud y de otros organismos del Poder Ejecutivo tales como oficios, memorandos y normativas emitidas mediante acuerdos ministeriales. Además, se realizó una búsqueda bibliográfica de fuentes secundarias a fin de obtener información sobre elementos conceptuales y de la experiencia de otros países para sustentar el análisis.

Se realizó una primera depuración de los documentos encontrados en la búsqueda a partir de los títulos y luego sobre la base del contenido de los resúmenes, utilizando como criterio de selección que el enfoque fuese la formación del recurso humano en salud, la del recurso humano en salud orientada a la atención primaria de salud (APS), y la reforma del sector salud. Tras la selección, en total se utilizaron 21 fuentes bibliográficas: fuentes primarias, entrevistas y fuentes secundarias.

## RESULTADOS

La revisión documental realizada permitió identificar los ejes temáticos en torno a los cuales se implementó la reforma en el desarrollo de los recursos humanos en salud en Ecuador. Dichos ejes se describen en los siguientes apartados. Los documentos encontrados en cada fuente bibliográfica se presentan en el cuadro 1.

### El desarrollo del recurso humano en salud para un nuevo modelo de atención

Bajo el mandato de la Constitución de Ecuador de 2008 (1) y de la Ley Orgánica de Salud del año 2006 (2), el MSP formuló un nuevo modelo de atención (el Modelo de Atención Integral de Salud o MAIS), que está basado en el marco de la APS (3). Con ello, el MSP hizo suya la definición de salud como pilar del desarrollo humano sostenible, entendiendo que el desarrollo de las comunidades se enmarca en el cuidado de su salud y que, a través de un enfoque de promoción y prevención, es posible alcanzar el desarrollo individual y colectivo al que aspira el país: *La salud y el enfoque de atención primaria en salud están en estrecha relación con la noción de desarrollo humano sustentable,… plantea el desarrollo como bienestar y generación de oportunidades en el presente y para las generaciones futuras *(4).

El MAIS planteó el cambio de enfoque desde lo curativo hacia lo promocional y preventivo, lo cual a su vez determina la necesidad de realizar modificaciones estructurales en la provisión de los servicios de salud y en el perfil de los profesionales que proveerán dichos servicios (5). Ello se plasmó en el desarrollo de carteras de servicios acordes con una nueva tipología de los establecimientos de salud (6) y en la definición de nuevos perfiles para el personal de salud, lo cual permitió identificar posteriormente la brecha del talento humano requerido. Con esta información, se creó el Programa de Becas para Fortalecimiento del Talento Humano en Salud (7), destinado a proveer fondos para promover la formación de profesionales de la salud en las especialidades con deficiencias.

**Cuadro 1.** Resultados de la búsqueda bibliográfica

Palabras clave	Bases de publicaciones		
	Publicaciones de la OPS	PubMed	ProQuest Health Management Database
Atención primaria en salud	175 000	355	203
Formación sanitaria	7 390	18	60
Reforma en salud/Health Reform	88 500	41 671	160
Recursos humanos en salud	50 500	28	151

***Fuente:*** Publicaciones de la OPS, PubMed y ProQuest Health Management Database.

De este modo, el trabajo conjunto del MSP, la Secretaría Nacional de Educación Superior, Ciencia, Tecnología e Innovación (SENESCYT), el Consejo de Educación Superior (CES), el Consejo de Evaluación, Acreditación y Aseguramiento de la Calidad de la Educación Superior (CEAACES), y el Instituto de Fomento del Talento Humano (IFTH), enfocado en la educación de los profesionales de la salud sobre la base de la APS, ha marcado un hito importante en la forma de *hacer salud* en Ecuador, y hace recaer un nuevo énfasis en especialidades que van más allá de lo asistencial, tales como la epidemiología, la antropología y la sociología, en concordancia con lo ocurrido en otros países de la Región de las Américas como Brasil y Cuba (5). Asimismo, en el marco del nuevo Modelo de Atención, la Autoridad Sanitaria respondió a las necesidades identificadas impulsando la formación de los técnicos de atención primaria en salud (TAPS) (8) y estimulando la formación de Médicos Familiares (9).

### La organización de estrategias formativas para los estudiantes y profesionales de las carreras de la salud

El panorama en años previos a 2012 era confuso en cuanto a la organización del recurso humano en salud. Según una entrevista realizada a la informante clave Dra. Cristina Merino, oficial a cargo de la cooperación técnica en desarrollo de recursos humanos para la salud en la Representación de las OPS/OMS en Ecuador, la insuficiente capacidad de planificación del MSP no permitía identificar ni proyectar las necesidades presentes y futuras del país en materia de personal de salud para cubrir las demandas de salud de la población, tanto en el ámbito asistencial como en lo relativo a funciones estratégicas de gestión y regulación del sistema en su conjunto. Las universidades definían sus mallas curriculares en carreras de la salud sin la orientación de la Autoridad Sanitaria (es decir el MSP), realizaban sus propios convenios para la formación de profesionales de la salud con los hospitales, y tomaban decisiones sobre el acceso a posgrados de los mismos (10). El MSP no asumía un papel rector en la definición ni en el desarrollo de la carrera de los profesionales de la salud y sólo se involucraba en la organización del año del servicio social de salud rural en calidad de prestador a fin de llenar vacantes en establecimientos de su propia red de provisión.

Frente a este panorama, y en el ámbito del proceso de reforma, se identificó la necesidad de fortalecer la rectoría del MSP en cuanto a la formación de los profesionales de la salud sobre la base de las necesidades nacionales y conforme a lo que establece la Ley Orgánica de Salud (2). A fin de emprender con éxito esta tarea, el MSP creó en 2012 la Dirección Nacional de Normatización del Talento Humano en Salud con la misión de *definir, normar y garantizar el cumplimiento de estándares relacionados con la planificación, gestión, formación y desarrollo del talento humano en Salud *(11).

A partir de la creación de esta nueva institución, ese mismo año se inició el diseño y la implementación de mecanismos administrativos y jurídicos destinados a definir los papeles y las relaciones entre los agentes institucionales que intervienen en el desarrollo del recurso humano en salud e institucionalizar los procesos de formación, reclutamiento y retención del personal en los establecimientos de salud del subsistema público. Esto implicó la articulación y el trabajo mancomunado con las instituciones rectoras de educación superior, así como la habilitación y acreditación profesional de la SENESCYT, por un lado, y con trabajo (Ministerio del Trabajo o MDT), por otro. Como parte de este proceso, el MSP formalizó su participación en la Comisión de Salud del CES y se firmó un convenio tripartito entre el MSP, la SENESCYT y el IFTH.

Lo anterior hizo posible desarrollar un conjunto de normas destinadas a regular la formación de los profesionales de la salud, como, entre otras, la Norma de Unidades Asistenciales Docentes (12), la Norma para acceder a programas de especialidades médicas (13) y el Reglamento para otorgamiento y devengo de becas (14). Estos instrumentos permitieron organizar la malla curricular de la carrera de medicina en pregrado y posgrado, conferirle un carácter no aleatorio y enfocarla hacia la satisfacción de las necesidades identificadas de los recursos humanos (15). Para implementar el posgrado, el MSP trabajó con nueve facultades de medicina a escala nacional y, por primera vez en el país, se estructuró un currículum único basado en las necesidades de la red pública de salud (16).

Asimismo, el MSP se abocó a resolver problemas específicos en el desarrollo del personal de salud ya inserto en el ámbito laboral, intensificando su relación con el MDT. Como resultado de estos esfuerzos, se publicó la Norma Conjunta de Internado Rotativo (17), se mantuvieron diálogos con los profesionales de la salud, se inició el trabajo para diferenciar horarios laborales y soluciones a las limitaciones de la Ley del Servidor Público para el personal de salud, y se concluyó afirmando que es necesario contar con una carrera sanitaria en Ecuador.

### Mejoría de la gestión de la formación de los profesionales de la salud

En el programa de educación de los profesionales de la salud se encuentra el pregrado, el posgrado y la formación continua. En lo referente al pregrado, el MSP concentró esfuerzos en lograr una mejor gestión de dos estrategias ya existentes, el Internado Rotativo y el año de Servicio de Salud Rural.

El Internado Rotativo es un requisito obligatorio del último año de la carrera de medicina, que consiste en que el estudiante acude regularmente a un establecimiento de salud para poner en práctica sus conocimientos teóricos bajo la tutoría de un profesional de la salud. Para organizar el Internado Rotativo, se definieron los requisitos que un establecimiento de salud debe reunir para poder desarrollar actividades docentes y adquirir la categoría de Unidad Asistencial Docente (UAD), de acuerdo con la norma creada a tal efecto (12). Los profesionales de la salud que realizan actividades de tutoría en las UAD reciben un sueldo por su labor asistencial y una remuneración adicional por sus actividades docentes otorgada por la universidad a la cual pertenecen los alumnos.

Por su parte, el año de Servicio de Salud Rural consiste en que los profesionales recién graduados ponen en práctica sus conocimientos asistenciales prestando atención de salud en diversos establecimientos a lo largo del país. Acorde con los principios del MAIS, este año de práctica obligatoria se reorientó hacia actividades de promoción y prevención en el primer nivel de atención de la Red Pública Integral de Salud (3). En la actualidad, el Ministerio de Salud Pública es quien asigna a los médicos rurales a los establecimientos de salud sobre la base de las brechas de necesidades de profesionales identificadas.

En lo relativo a la educación de posgrado, la Autoridad Sanitaria Nacional estableció, en el marco del Proyecto de Fortalecimiento del Talento Humano, un fondo sin precedentes para otorgar becas de posgrado en el extranjero y estimular la formación en especialidades para las cuales se habían identificado deficiencias en la red asistencial del MSP. Entre 2013 y 2015 se concedieron becas por un total de alrededor de $US 31 millones con esta finalidad. (La cifra exacta, según las células presupuestarias para 2015 del Proyecto de formación de talento humano del MSP, asciende a $US 30.881.590,42.) El Reglamento para otorgamiento y devengameinto de becas establece que los becados deberán devengar la beca en los establecimientos de salud del Ministerio con el objetivo de retribuir al país el beneficio recibido. Se espera que esta estrategia permita resolver la brecha de especialistas según las necesidades de la población. La asignación de becas y el devengamiento de las mismas reflejan una mejor organización, la incorporación de una visión de futuro y una planificación más integral de los recursos humanos en salud por parte de la Autoridad Sanitaria.

### Estrategias de desarrollo laboral para los profesionales de la salud

La problemática laboral de los profesionales de la salud había sido abandonada históricamente por el ente rector sin brindar soluciones visibles a sus demandas. Como bien se menciona en el Informe sobre la Salud en el Mundo de la OMS de 2006, es necesario que el recurso humano reciba una remuneración ajustada a su trabajo y esfuerzo, pues este afecta la calidad del servicio que otorga (18). Por este motivo, la Autoridad Sanitaria, enfocada a brindar soluciones en el desarrollo laboral del personal de sanitario, identificó la necesidad de disponer de incentivos tanto económicos como de orden general, que motiven al personal, ya que se ha comprobado que los incentivos suelen ser más eficaces cuando incluyen un grupo de beneficios (18).

Antes de la reforma, los profesionales sanitarios del subsistema público en Ecuador contaban con una remuneración que no era competitiva con el salario ni con los incentivos que se ofrecían en el sector privado. En este área, el MSP realizó un esfuerzo importante para mejorar la situación laboral del personal sanitario. En relación con los médicos, su salario aumentó aproximadamente 80% cuando se trabajó en una homologación de salarios en el sector público y se estipularon incentivos a zonas de difícil acceso para prevenir la migración de profesionales (16, 19–21).

El aumento de salarios de los profesionales de la salud fue uno de los objetivos iniciales que tuvo mayor resonancia, puesto que no benefició únicamente a los médicos, sino también a enfermeras y profesionales de la asistencia sanitaria. Como se sostiene firmemente que el per-sonal sanitario es un pilar para reforzar los sistemas de salud y que se debe invertir en ellos (18), este trabajo se ve reflejado en la homologación de los salarios de los servidores públicos (19). Con una mejor remuneración se pudo dar inicio a un trabajo complejo relativo al diseño e im-plantación de incentivos adicionales para los profesionales sanitarios.

La remuneración homologada cumplió con una finalidad adicional al aumento salarial: evitar la migración de los profesionales dentro del país hacia las ciudades más grandes (16). Conociendo la importancia de un despliegue eficaz de los trabajadores sanitarios (18), el MSP quiso garantizar la disponibilidad de profesionales de calidad en todo el territorio nacional, aportando así recursos al correcto funcionamiento del MAIS. Junto con un salario homologado, se identificó que existían áreas de difícil acceso en las cuales se necesitaba trabajar con el MDT para motivar a los profesionales de la salud a ofrecer sus servicios en ellas. Por tal motivo surgió la Norma para bonificación geográfica a ser-vidores del sector salud (21), que plantea bonificaciones a las áreas determinadas de difícil acceso, mejorando así la cobertura nacional de profesionales de la salud.

Para cubrir la necesidad de profesionales sanitarios preparados es importante prever adicionalmente su etapa de inser-ción al sistema laboral. Se debe asegurar un entorno laboral favorable (5), y en esta línea el MSP formuló una normativa de apoyo y mejora de la situación de estudios y laboral del Interno Rotativo. El Internado Rotativo en Ecuador tiene un salario que previamente se negociaba aleatoriamente en ausencia de normas. El MSP, junto con el MDT, elaboró la Norma Conjunta de Internado Rotativo (17), en la cual se menciona claramente el horario del interno, su salario y se convierte en obligatoria su afiliación al Instituto Ecuatoriano de Seguridad Social (IESS), un beneficio laboral adicional para su correcto desempeño.

### La carrera sanitaria como pilar de desarrollo del recurso humano en salud

La carrera sanitaria se definió como una necesidad del país, reconocida y solicitada por los profesionales de la salud y priorizada por la Autoridad Sanitaria. De la información disponible se puede inferir que, durante los años de la reforma del sistema de salud, varios de los problemas que encontró el MSP en su trabajo respecto al recurso humano en salud se deben a que Ecuador carece de una carrera sanitaria que permita el desarrollo integral del personal de salud.

Uno de los ejemplos más tangibles en la actualidad sobre este problema, que ha surgido al no tener Carrera Sanitaria, se puede identificar al tratar temas laborales, ya que el profesional que trabaja en el sector público en Ecuador se rige por la Ley Orgánica del Servidor Público (19). Los profesionales contratados en los establecimientos de salud del MSP tienen un contrato llamado ocasional, que limita la continuidad del personal de salud en el trabajo solamente a dos años. Este tipo de contrato impide cumplir con el plan de capacitación al profesional de salud, a pesar de que la capacitación es un incentivo y una motivación para el personal. Los profesionales de la salud que trabajan en el subsistema de salud público luchan por trabajar bajo otra figura de contrato que les permita desarrollarse apoyando a las unidades con su experiencia acumulada durante varios años y por tener la oportunidad de acceder a la formación continuada necesaria como parte de una carrera sanitaria.

Como se menciona en el Informe sobre la Salud en el Mundo 2006, *Las estrategias nacionales de desarrollo del personal sanitario deben mirar más allá de los salarios y la formación en el sector público para abarcar todo el ciclo de entrada – etapa laboral – salida tanto en el sector privado como en el público. El desarrollo de la fuerza laboral es una labor a la vez técnica y política, y exige generar confianza entre los interesados y vincular las expectativas de las personas al desempeño de los profesionales*(18).

En concordancia con lo mencionado, el MSP desarrolla una estrategia integral, con la cual se intenta tener en cuenta todos los esfuerzos realizados por los profesionales de la salud en sus estudios de pregrado, la remuneración en el trabajo, los estudios de posgrado, los años de experiencia en el trabajo y la formación continua, y validarlos a través de la Carrera Sanitaria. Esta es una deuda de la reforma del período 2012–2015 con el país, que deberá consolidarse durante el presente año, pues, como declaró un entrevistado clave, el economista Andrés Egas, Director Nacional de Talento Humano en Salud, hasta la actualidad se mantiene el compromiso de publicar la Carrera Sanitaria en el año 2016 (15).

## DISCUSIÓN

El componente de desarrollo de recursos humanos del proceso de reforma en salud de Ecuador en el periodo 2012–2015 alcanzó importantes logros gracias al trabajo intersectorial realizado por el MSP y el fortalecimiento de la rectoría para comprender y gobernar el Sistema de Salud y lograr crear nuevos perfiles de profesionales, normativas, regulación y la cobertura nacional de profesionales para implementar el MAIS, que resolvió problemas arrastrados durante los años previos a la reforma.

Durante el desarrollo de este trabajo, se identificó claramente la necesidad de disponer de una plataforma única que sustente todas las normativas en forma de una carrera sanitaria. Por esta razón, se puede sugerir a otros países que se encuentren en el proceso de la reforma que desarrollen la carrera sanitaria como punto de partida, lo cual puede conferir mayor fluidez al desarrollo de la normativa adicional y de regulaciones que se consideren necesarias.

Además, se observa que los esfuerzos se concentraron en la generación de estrategias de desarrollo para el personal sanitario asistencial y que no hubo un desarrollo paralelo de estrategias dirigidas al personal sanitario que realiza funciones estratégicas tales como la formulación de políticas, la planificación, el desarrollo normativo, la gerencia, la gestión del suministro de medicamentos y dispositivos médicos, y la vigilancia epidemiológica. En efecto, no se consiguió identificar estrategias para desarrollar los perfiles profesionales necesarios para formular e implementar los diversos elementos de la política de salud en Ecuador.

Asimismo, este estudio muestra que, a pesar de los esfuerzos realizados por el MSP para ejercer la rectoría sobre el sistema de salud, la visión predominante sigue siendo fragmentada y estando centrada en la red asistencial propia, con una clara priorización del desarrollo de recursos humanos para la provisión de servicios de salud en los establecimientos del MSP. Una clara muestra de ello es que el MSP destinó esfuerzos para reducir brechas de profesionales únicamente en sus unidades de salud omitiendo a la Red Pública Integral y a la Red Complementaria. A este respecto, es importante continuar fortaleciendo el papel rector y realizar en los próximos años un estudio de brechas de profesionales que contem-ple las redes de provisión de la Seguridad Social y el sector privado.

Finalmente, cabe destacar que el trabajo realizado en la reforma 2012–2015 sentó las bases que permitirán a Ecuador dar un salto cualitativo en la estimación, contratación y desarrollo de los recursos humanos necesarios en salud, al incorporar elementos relacionados con la demanda de servicios de salud, infraestructura, cartera de servicios y personal requerido en los próximos años en función de los cambios estimados del perfil epidemiológico del país. Esto puede constitutir una aportación fundamental a la consolidación de la planificación sanitaria de los recursos humanos como función esencial de salud pública.

## Agradecimientos.

Los autores agradecen a Cristina Merino de la Organización Panamericana de la Salud todo su apoyo durante la redacción del artículo y la valiosa información brindada al equipo de autores.

## Declaración.

Las opiniones expresadas por los autores son de su exclusiva responsabilidad y no reflejan necesariamente los criterios ni la política de la Organización Panamericana de la Salud o de la *RPSP/PAJPH*.
